# Immunomodulatory potential of primary cilia in the skin

**DOI:** 10.3389/fimmu.2024.1456875

**Published:** 2024-11-29

**Authors:** Jingwei Sun, Huimin Yuan, Yanru Yu, Aorou Li, Zihe Zhao, Yang Tang, Fengjie Zheng

**Affiliations:** ^1^ School of Traditional Chinese Medicine, Beijing University of Chinese Medicine, Beijing, China; ^2^ Department of Dermatology, Beijing Tsinghua Changgung Hospital, School of Clinical Medicine, Tsinghua University, Beijing, China

**Keywords:** primary cilia, skin, signaling pathway, immunoregulation, keratinocytes, Langerhans cells, fibroblasts

## Abstract

Primary cilia (PC) are essential signaling hubs for proper epithelial formation and the maintenance of skin homeostasis. Found on most cells in the human body, including skin cells, PC facilitate signal transduction that allows ciliated cells to interact with the immune system via multiple pathways, helping to maintain immune system homeostasis. PC can be altered by various microenvironmental stimuli to develop corresponding regulatory functions. Both PC and ciliary signaling pathways have been shown to be involved in the immune processes of various skin lesions. However, the mechanisms by which PC regulate cellular functions and maintain immune homeostasis in tissues are highly complex, and our understanding of them in the skin remains limited. In this paper, we discuss key ciliary signaling pathways and ciliated cells in the skin, with a focus on their immunomodulatory functions. We have compiled evidence from various cells, tissues and disease models to help explore the potential immunomodulatory effects of PC in the skin and their molecular mechanisms.

## 1 Introduction

The skin serves as a life-sustaining interface between the body and the environment, functioning both as a good mechanical barrier and as the body's first line of immune defense. Extensive interactions between epithelial, stromal, and immune cells regulate the skin's immune response (1). Immune dysregulation in the skin leads to localized inflammatory infiltration and impaired epithelial function, triggering a variety of inflammatory skin diseases. These conditions are primarily mediated by T cells, the humoral immune system, or nonspecific inflammation (2), each involving distinct immune response patterns (3).

Primary cilia (PC) are highly conserved organelles protruding from the cell surface. They present abundant receptors, ion channels, and downstream effectors for multiple signaling pathways (4). PC can perceive and transmit extracellular signals to regulate various cellular processes during development and to maintain tissue homeostasis. PC are dynamic structures formed from the centriole. Since centrioles serve a dual function in spatially organizing the mitotic spindle and forming cilia, the genesis and disassembly of cilia are inextricably linked to cell cycle progression (5–7).

Numerous studies have shown that keratinocytes (KC), Langerhans cells (LC), melanocytes and fibroblasts of the skin express PC (8–11). During skin growth and development, approximately 60–75% of epidermal cells express PC (8). Structural or functional ciliary defects can lead to a variety of sensory, physiological, and developmental abnormalities (12). Damage to PC poses threats to the normal growth and differentiation, tissue homeostasis, and barrier function of the skin (8, 13–15). Studies have shown that PC may contribute significantly to skin inflammatory processes such as skin wound healing and scarring (13). The epidermis of patients with inflammatory skin diseases such as atopic dermatitis and psoriasis expresses high numbers of ciliated cells (9, 16).

PC are relatively understudied organelles in the skin. Beyond their role in integrating extracellular signaling pathways, they also modulate the activation of multiple inflammatory signals and the expression of pro-inflammatory cytokines (17–22). Another prospective viewpoint suggests that PC may regulate the number, phenotype, and function of immune cells through noncell-autonomous mechanisms; epithelial ciliary signaling may modulate the behavior and function of non-ciliated immune cells, leading to inflammatory responses (23, 24). Despite accumulating evidence revealing that PC have an important regulatory role in immune system function, the immunomodulatory role and molecular mechanisms of PC in the skin remain largely unexplored (25). This review aims to examine the immunomodulatory potential of PC in the skin based on the current knowledge of their immunomodulatory function.

## 2 Signal transduction function of primary cilia

**Figure 1:**
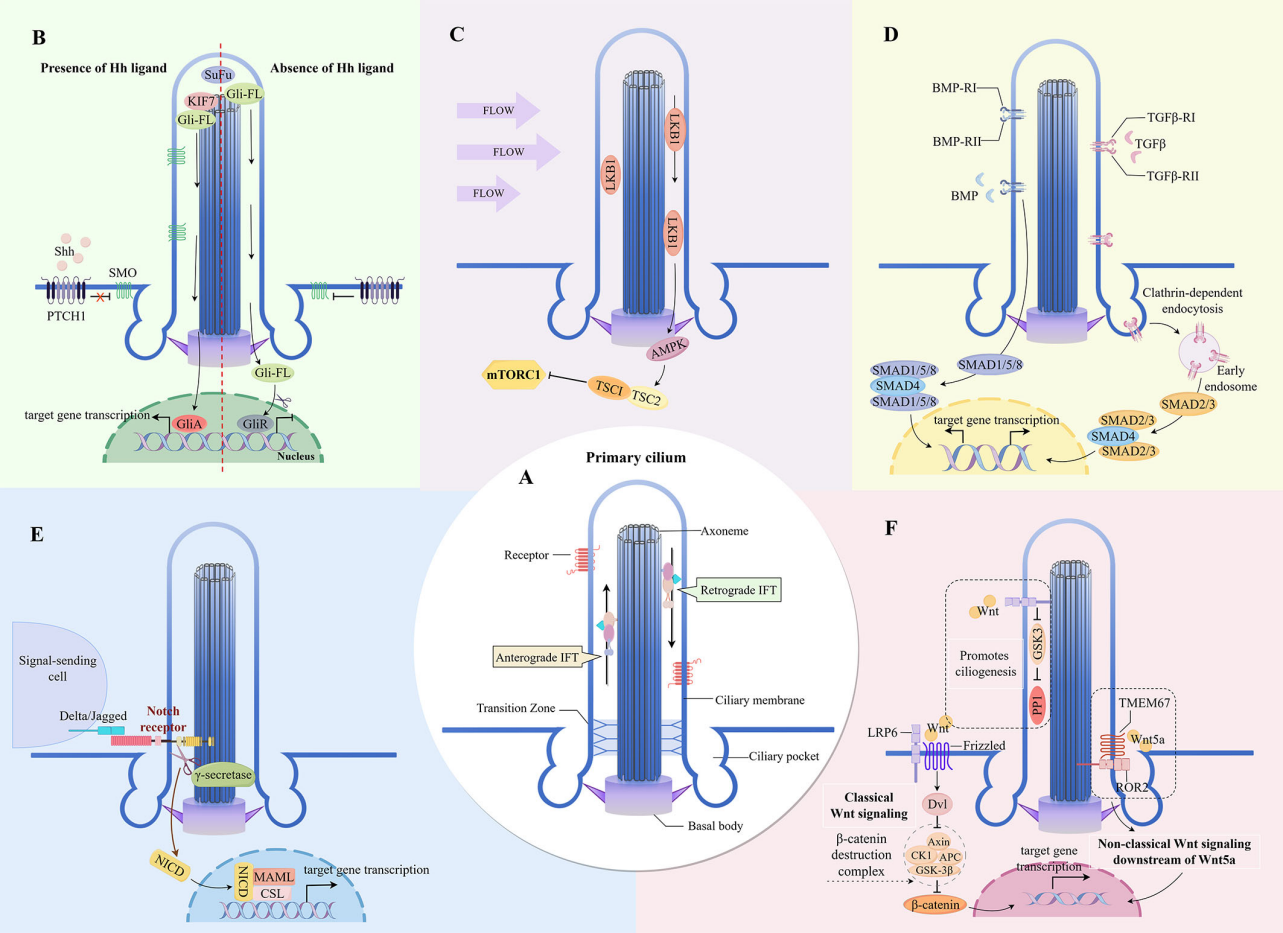
Key signaling pathways mediated by primary cilia in the skin **(A)** The major structures of primary cilium. **(B)** Cilia-dependent Hedgehog signaling. **(C)** Cilia-dependent mTOR signaling. **(D)** Cilia-dependent TGF-β/BMP signaling. **(E)** Cilia-dependent Notch signaling. **(F)** Cilia-dependent Wnt signaling. IFT, intraflagellar transport; SOM, Smoothened; PTCH1, Patched-1; SUFU, Suppressor of Fused; Gli-FL, full-length versions of GLI transcription factors; GliA, Gli activator; GliR, Gli repressor; LKB1, liver kinase B1; AMPK, AMP-activated protein kinase; TSC1/2, Tuberous sclerosis complex 1/2; SMAD, Small mother against decapentaplegic protein transcription factor; NICD, Notch intracellular domain; MAML, Mastermind-like; LRP6, low-density-lipoprotein-receptor-related protein 6; Dvl, Dishevelled; GSK3-β, glycogen synthase kinase 3 beta; PP1, phosphatase 1; TMEM67, transmembrane protein 67; ROR2, receptor tyrosine kinase-like orphan receptor 2.

PC are microtubule-based organelles that extend from the basal body (centriole) and protrude from the cell surface (4). The ciliary membrane is continuous with the plasma membrane, overlying the axoneme (4). The ciliary proteins required to maintain ciliogenesis and function must be transported from the cytoplasm, where they are generated, a process that relies on intraflagellar transport (IFT) (26, 27). IFT particles move bidirectionally along the axonemal microtubules between the base and tip of the cilium for the transport of structural and signaling components. Changes in ciliary length usually indicate altered ciliary function (28). The unique ciliary structure provides a suitable space for signal transduction. It includes ciliary membranes enriched in specific transmembrane receptors and signaling functional lipids, ciliary pockets highly capable of endocytosing signaling molecules, ciliary cytoplasm enriched in second messengers and effector proteins, transition zones controlling the ciliary entrance and exit of soluble and membrane-bound signaling molecules, and IFT mediating the transport of membrane-bound and soluble signaling proteins (4, 29) (**Figure 1**). The structure and composition of PC enable them to receive, transmit and integrate biological information and play an important role as signaling hubs in intercellular communication. Primary cilium structural and functional abnormalities usually lead to dysfunctions in signaling and cellular function, resulting in polymorphic diseases and syndromes (12).

In skin lesions, the signaling properties of PC are associated with various immunoregulatory functions. The role of PC in the tumor immune microenvironment, particularly their regulation of immune cells, has garnered significant attention (24). Studies have shown that PC-dependent signaling pathways are involved in the pathogenesis of skin cancers such as basal cell carcinoma [dependent on the PC-activated Hedgehog (Hh) pathway (30)] and melanoma [dependent on the PC-inhibited Wnt signaling (31)]. Targeted inhibition of the Hh pathway for basal cell carcinoma treatment notably alters the immune microenvironment of basal cell carcinoma, promoting adaptive immune reactions via upregulation of major histocompatibility complex (MHC) class I and cytotoxic T cells recruitment, alongside a reduction in PC (30). Additionally, PC antagonize the conversion of Hh to the Ras/MAPK pathway, which may explain why PC loss leads to resistant basal cell carcinoma (32). Research has also found that oxidative stress in NRF2-downregulated melasma keratinocytes inhibits ciliogenesis and Hh signaling, which in turn promotes keratinocyte differentiation, melanin synthesis, and melanosome transfer to keratinocytes (33). Dermal lymphatic endothelial cells are also found to possess PC. It has been suggested that PC-dependent signaling may have a regulatory function in maintaining lymphatic vessel homeostasis as well as various lymphatic vessel patterns in inflammation, wound healing, recurrent inflammation, and tumor microenvironment (34). The contribution of PC signaling in inflammatory processes of skin wound repair and healing has also been summarized (13). Cilia-dependent signaling pathways are likely involved in characteristic skin inflammatory processes such as skin thickening, fibrosis, immune cells activation and infiltration.

## 3 Key signaling pathways mediated by primary cilia in the skin

### 3.1 Hedgehog signaling pathway

The vertebrate Hh signaling, a typical signaling pathway transduced by PC, is absolutely dependent on these organelles (35) (**Figure 1**). The Hh signaling pathway is a significant regulator of skin inflammation, specific immune responses, and the skin barrier function (36). In melasma, inhibition of ciliogenesis and Hh signaling in KCs stimulates KC differentiation, leading to skin hyperpigmentation (33). Inturned (INTU), a ciliogenesis and planar cell polarity effector protein, is essential for the formation of PC in the skin. The loss of epidermal PC due to INTU protein disruption causes inhibition of Hh signaling and results in aberrant differentiation of follicular KCs (37). Conversely, INTU disruption in basal cell carcinoma with highly expressed ciliated cells and Hh signaling is effective in controlling disease progression (38).

Sonic hedgehog (Shh) is the major ligand for Hh in the skin. The presence or absence of ligands such as Shh controls the level of enrichment of the transmembrane protein receptor Patched-1 and the seven-transmembrane protein Smoothened on cilia, as well as the activation of Hh signaling (39). In atopic dermatitis, both activation of the Hh pathway in skin T cells and Shh expression are increased, and upregulation of Shh signaling attenuates the pathological alterations of the disease (40). Gli transcription factors are endpoints of the Hh pathway that bind to DNA at consensus Gli-binding sites to repress or promote the expression of Hh-targeted genes (40). PC and ciliary proteins such as Kif7 regulate the conversion of Gli to either its repressive or its activated form (35, 41).

### 3.2 mTOR signaling pathway

mTOR is an atypical serine/threonine protein kinase that forms two distinct signal transduction complexes, mTOR complex 1 (mTORC1) and mTOR complex 2, by binding to a variety of concomitant proteins. The two kinase complexes are differentially sensitive to upstream input and downstream output signals, and they have specific regulatory functions on cells (42). The regulation of KC proliferation, epidermal stratification, and hair follicle formation by mTOR signaling is critical for normal skin morphogenesis and epidermal barrier formation (43). mTOR signaling pathway activation can downregulate the expression of epidermal barrier-associated proteins (44, 45).

The proteins and signaling molecules residing in PC may regulate mTORC1 activity (**Figure 1**). The negative regulator of mTOR, Lkb1, is enriched in cilia and basal bodies (78). Flow stress-induced bending of cilia activates the Lkb1 pathway in the basal region, resulting in a large accumulation of phosphorylated AMPK at the basal body (46). Tuberous sclerosis complex (TSC) 1 and TSC2 are key regulators of mTORC1. Activated AMPK forms the TSC1/TSC2 complex by phosphorylating TSC2; TSC1/TSC2 can stimulate the GTPase-activating protein of TSC2 to bind to Rheb (Ras homolog enriched in brain) and suppress mTORC1 activity (47).

In terms of immune regulation, mTOR is a critical signaling node that senses the immune microenvironment and integrates immune signals and metabolic processes that determine the maintenance and activation of T cells (48–50). mTOR is commonly upregulated in inflammatory skin diseases (51). PI3K/Akt is the classical upstream pathway of mTOR (49), and the PI3K/Akt/mTOR signaling cascade is critical in the development of immune-mediated inflammatory skin diseases. For example, in psoriasis, hyperactive PI3K/Akt/mTOR1 signaling leads to hyperproliferation of KCs and increased cellular inflammation (52, 53). mTOR inhibition effectively alleviates excessive immune responses (54). In addition, autophagy is a key downstream event of mTORC1 (55). Autophagy is a key mechanism for organelle degradation during the terminal differentiation of epidermal KCs (56), and a lack of autophagy impairs the epidermal barrier and exacerbates inflammation (56–58). A reciprocal positive interaction between PC and autophagy exists, where mTOR signaling is activated in cilia-suppressed (cilia-shortened) cells and results in autophagy inhibition (59). However, the exact mechanism of mTOR signaling by skin cilia remains to be clarified.

### 3.3 Wnt signaling pathway

The Wnt signaling pathway is a complex regulatory network with three main branches: the classical Wnt/β-catenin pathway, and the two main non-classical Wnt pathways induced by Wnt family member 5A (Wnt5a), the Wnt/planar cell polarity and Wnt/Ca2^+^ signaling pathways (60, 61). Proinflammatory functions of Wnt5a trigger proinflammatory signaling cascades that increase the secretion of proinflammatory cytokines and chemokines (62). Both Wnt5a and its receptor (Frizzled) are redistributed in psoriatic lesions (63–65) and overexpressed throughout all epidermal layers of the lesions, inhibiting downstream β-catenin signaling in the classical Wnt pathway (63, 65). This process involves Wnt5a-regulated disruption of KC proliferation and differentiation, as well as a series of Wnt5a-induced proinflammatory signals (63, 64, 66).

Research indicates that kinases, phosphatases, and proteasome proteins involved in signaling between PC and Wnt pathway members are primarily localized in the basal and transition zones of the cilium (67, 68). This localization allows the classical and non-classical Wnt signaling pathways to converge at the basal body (67, 68). In the ciliary transition zone, the atypical Wnt signaling receptor transmembrane protein 67 binds Wnt5a and acts as a co-receptor to mediate downstream signaling via ROR2 (69). As a result, the pro-inflammatory action of Wnt5a in skin inflammation may depend on the signaling at the cilia (**Figure 1**). Additionally, PC can also respond directly to endogenous and exogenous Wnt signals without traversing through the nonciliated regions of the plasma membrane and the cytoplasm, promoting ciliogenesis through the b-catenin-independent WNT/GSK3 pathway (70). Furthermore, PC are not only organelles that transduce Wnt signaling, but their assembly and disassembly are regulated by the Wnt signaling pathway (71).

### 3.4 TGF-β/BMP signaling pathway

The transforming growth factor β (TGF-β) superfamily of proteins consists of subfamilies such as TGF-β and bone morphogenetic protein (BMP). PC are important coordination sites for TGF-β signaling (**Figure 1**), and the receptors for TGF-β signaling, TGFβ-RI and TGFβ-RII, are localized at the tip and base of the cilia (72). BMP signaling is similarly regulated by signaling proteins that are localized in PC (73, 74). Upon ligand binding, the TGF-β receptor translocates to the ciliary pocket, where it gets internalized by clathrin-dependent endocytosis, forming clathrin-coated vesicles and early endosomes (72). Small mother against decapentaplegic (SMAD) protein transcription factors, SMAD2/3 (downstream of TGF-β signaling) and SMAD1/5/8 (downstream of BMP signaling) (74) are phosphorylated by early endosomal anchoring. Activated SMAD transcription factors form a trimeric complex with an auxiliary SMAD (SMAD4) and translocate to the nucleus for the expression of target genes (75).

TGF-β1 is the most abundant TGF-β isoform in most tissues, including the skin (76). TGF-β1 has been shown to have immunosuppressive effects (77); however, its role in the development and migration of epidermal LCs (78, 79), as well as in the induction of T helper (Th) 17 cell differentiation (80, 81), highlight the pro-inflammatory effects of TGF-β1. Thus, TGFb1’s immunosuppression in the skin may be easier to overcome than that in other organs (82). In psoriatic epidermis lesions, typical TGF-β signaling levels are downregulated and TGF-β receptors are markedly diminished (83, 84), whereas increased TGF-β1 in the epidermis and serum is directly correlated with the severity of the skin inflammation (82, 85). This may be due to the enhanced production of molecules required for the development of Th1 inflammatory dermatopathies induced by the overexpressed TGF-β1 (82). Studies have shown that active TGF-β1 release promotes a variety of physiological or pathological processes via primary cilium-mediated TGF-β signaling; and, the absence of PC impairs functions that are dependent on TGF-β1 signaling (86, 87).

The typical TGF-β signaling molecules are expressed at low levels, but KC-derived BMP7 signaling is highly expressed throughout the proliferating psoriatic epidermis (88). TGF-β family members induce differentiation and proliferation of bone marrow-derived epidermal LC-like cells with a phenotype similar to LCs by preferentially activating the BMP signaling cascade (88) and enhance the stimulatory activity of Treg cells in inflammation (89). In addition, TGF-β signaling is also necessary for the formation and survival of tissue resident memory T cells (90, 91).

### 3.5 Notch signaling pathway

Notch signaling is involved in the normal epidermal proliferation and differentiation program. Notch signaling is upregulated in suprabasal cells and hair follicles, and Notch receptor expression sites coincide with cells that are initiating or undergoing terminal differentiation (92, 93). Notch signaling balances the proliferation and differentiation of KCs and LCs (94–97) and maintains the epidermal skin barrier function (93, 98). The Notch receptors (Notch 2, Notch 3) and the catalytic subunit of the protease γ-secretase localize in the epidermal cilia (8, 99) (**Figure 1**). Upon binding of the extracellular domain of the receptor to the Notch ligand on the neighboring cell membrane, the Notch receptor undergoes two proteolytic cleavages that release its intracellular domain (92). The Notch intracellular domain translocates to the nucleus and activates the expression of downstream target genes by binding to the transcriptional repressor CSL to form a complex and recruit the coactivator protein Mastermind (100, 101). In addition, PC can activate Notch signaling by translating the perceived shear stress (102).

Absence of PC attenuates Notch signaling and directly impairs epidermal differentiation and the skin barrier function (8). Notch dysregulation is an important phenotype in inflammatory skin diseases (103). The expression of Notch-related genes is downregulated and epidermal KC differentiation is inhibited in psoriasis skin biopsies (95, 104). Expression of Notch receptors is similarly markedly downregulated, or even undetectable, in the epidermis of patients with atopic dermatitis (105). The Notch signaling pathway also reinforces Th cell differentiation, particularly during the differentiation of CD4^+^ T cells to effector subpopulations such as Th1, Th2, and Th17 (106–108). Although the transduction function of PC in response to Notch signaling is crucial for skin development, the manifestation of PC that changes with Notch signaling in skin inflammation requires further study.

## 4 Involvement of primary cilia in the inflammatory response of skin-associated structures

### 4.1 Keratinocytes

KCs are the predominant cell type in the epidermis. Their proliferation begins in the basal layer of the epidermis, they gradually migrate, differentiate, and mature upward through the basal, spinosum, granulosum, and corneum strata, maintaining the normal structure and function of the epidermis (109). The expression of PC is associated with the proliferation and differentiation of KCs. Ciliogenesis may be negatively correlated with KC proliferation (8, 15). However, the increased numbers of ciliated cells in the epidermis of individuals with atopic dermatitis may interfere with the differentiation of the cells under the stress induced by tissue inflammation, leading to the overproliferation of immature LCs and KCs in the epidermis (9, 16).

**Figure 2:**
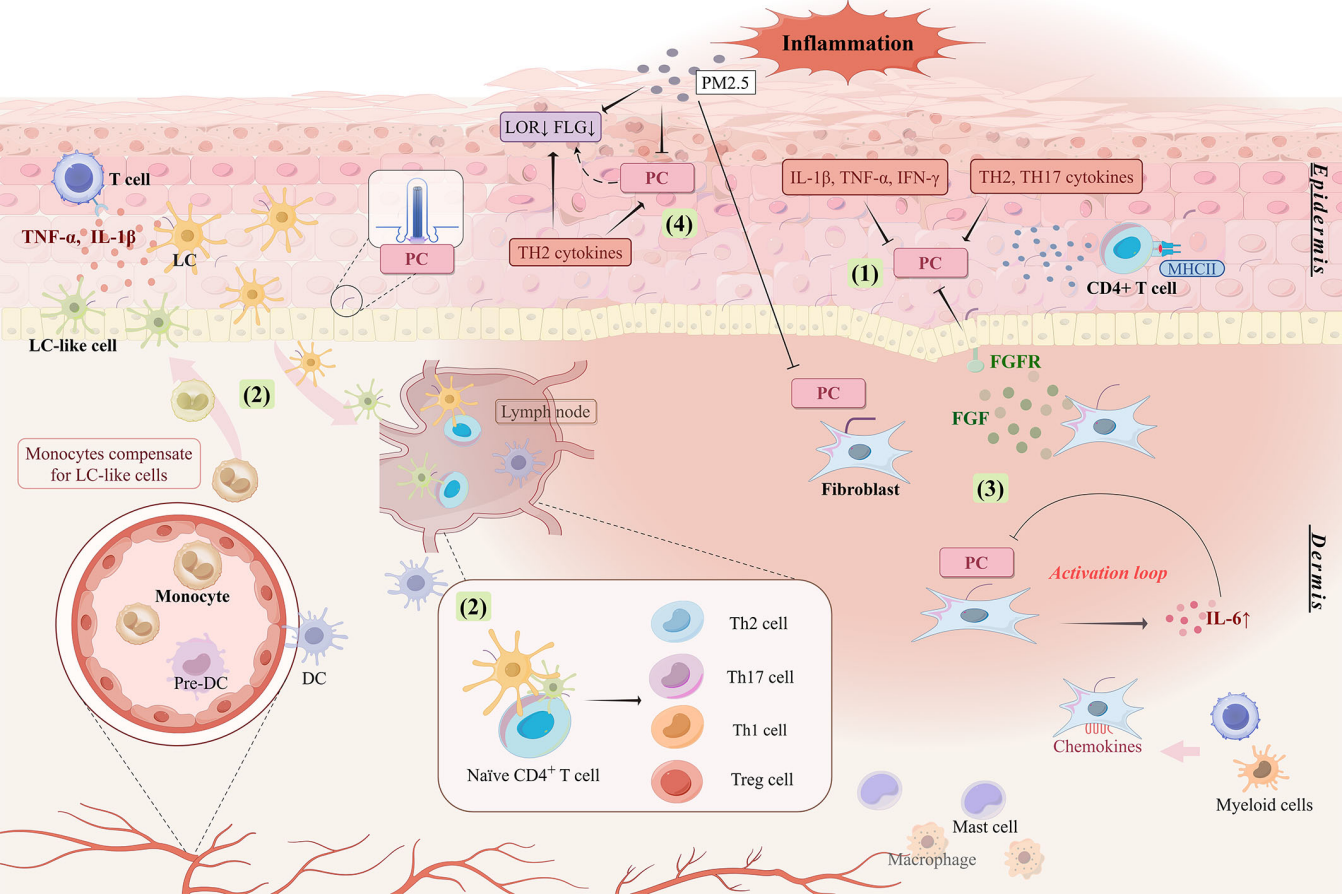
Involvement of ciliated cells in skin inflammatory responses. (1) Pro-inflammatory factors and TH2 and TH17 cytokines stimulate the aberrant expression of PC, which is manifested by changes in length and number. Abnormal expression of PC mediates the abnormal proliferation and differentiation of keratinocytes and promotes immune response and inflammatory process in the skin. (2) PC may be involved in regulating the niche of LC in inflamed skin to ensure the maintenance of LC number in the epidermis and to induce differentiation and response patterns of different T lymphocyte subsets. (3) The inflammatory response is amplified by the activation loop formed between the absence of PC on fibroblasts and the upregulation of IL6. High expression of fibroblast growth factor induces proliferation and abnormal differentiation of epidermal keratinocytes, which may be associated with the expression of PC in keratinocytes. (4) PC may be associated with low expression of skin barrier related proteins like LOR and FLG. PC are also involved in skin inflammation stimulated by external environmental triggers like PM2.5. PC, primary cilia; LC, Langerhans cell; DC, dendritic cell; FLG, filaggrin; LOR, loricrin; FGF, fibroblast growth factor; FGFR, fibroblast growth factor receptor; MHCII, major histocompatibility complex class II.

KCs constitute the mechanical barrier of the skin and have important immune functions. They express a range of pattern recognition and cytokine receptors that sense the immune environment, allowing them to respond to and express or secrete a wide range of pro-inflammatory cytokines, chemokines, and growth factors (110). These responses direct and activate most polarized immune responses, including Th1, Th2, Th17, and a variety of autoinflammatory responses, making KCs the causative agent of many, if not all, inflammatory skin disorders (110). The formation of PC in KCs is regulated by immune signaling stimuli. The number of ciliated cells increases significantly (without significant ciliary length changes) after stimulation of neonatal normal human epidermal keratinocytes with Th2 cytokines (interleukin [IL]-4, IL-13, and IL-31) and Th17 cytokines (IL-17A and IL-22), whereas the number of ciliated cells tends to decrease when stimulated with high concentrations of proinflammatory factors (IL-1β, tumor necrosis factor-alpha (TNF-α), and interferon-gamma (IFN-γ)) (16). Patients with atopic dermatitis and psoriasis have a significant increase in the number of PC expressed by KCs, which may be related to JNK phosphorylation regulated by the expression of the ciliary transport protein IFT88 (16). Dysregulation of the JNK pathway influences the onset of inflammatory dermatoses (111). Thus, KCs exhibit alterations in their primary cilium numbers while mediating diverse skin immune responses, and the immune function of KCs may be influenced by the sensing and transduction functions of their PC (**Figure 2**).

Upon activation by local skin inflammatory signals, KCs express MHCII molecules on their surface, allowing them to act as antigen-presenting cells (APCs) to activate localized (auto-reactive) CD4^+^ and CD8^+^ T cells (112–114) and induce Th1-based immune responses (113, 114). PC also appear to be involved in MHCII expression. Defects in the ciliary kinesin motor protein Kif7 reduce cell surface MHCII expression on thymic epithelial cells and seem to correlate with the overall activation level of the Hh pathway (115). Therefore, the expression of PC on KCs may influence the activation of local inflammatory signals on MHCII expression on KCs and the ensuing immune responses.

### 4.2 Langerhans cells

LCs are important cutaneous APCs, their migration to lymph nodes induces T cell differentiation in specific immune environments (94, 116–118). Different LC subpopulations synergistically modulate the homeostasis of the normal human skin and immune responses in diseases (94). LCs have been reported to proliferate extensively in the epidermis of inflamed skin (94, 119). In the epidermis of patients with atopic dermatitis, the expression of PC on LCs is increased with hyperproliferation of immature LCs (9). Studies on the exact mechanisms by which primary cilium expression affects LC function are lacking, but ciliary signaling pathways, such as TGF-β/BMP and Notch signaling, are known to mediate the inflammatory proliferation and differentiation of LCs. Thus, PC may influence LC function in skin inflammation via these pathways.

TGF-β1 is essential for the development and maintenance of epidermal LCs (78, 120). Disruption of TGF-β1 signaling drives LC migration from the epidermis to subcutaneous draining lymph nodes in both homeostatic and inflammation-inducing environments (121). Monocytes are also an essential source of LCs. Under inflammatory or injured conditions, monocyte-derived LC-like cells are rapidly recruited to the site of inflammation, activating skin T cells by producing inflammatory cytokines and amplifying inflammation (122, 123). *In vitro*, both TGF-β1 and BMP7 can induce the complementary differentiation of monocytes into LCs, but BMP7-induced LCs produce larger amounts of proinflammatory cytokines and exhibit a greater capacity to stimulate T cells than TGF-β1-induced LCs (88, 124). Notch signaling also mediates the generation of monocyte-derived LCs (96, 97) and promotes the differentiation of LC2 subpopulations with immunomodulatory effects (94). Cilia-associated signaling derived from epidermal KC is known to guide the self-renewal and differentiation of inflammation-associated LCs (88), suggesting that cilia-mediated signaling may be a key factor in facilitating the crosstalk between skin KCs and LCs.

Antigen presentation by LCs is one of the prerequisites for inducing T cells to initiate an immune response. In inflammatory environments, the number of LCs in the skin increases (derived from monocytes and/or skin-residents, but mainly from recruited monocytes) (122, 125) (**Figure 2**). LCs and macrophages share a common precursor; therefore, LCs share many similarities with tissue-resident macrophages in self-renewal maintenance and ontogeny (125, 126). Deletion of primary cilium component genes affects the macrophage niche, thereby contributing to an accumulation of infiltrating macrophages and a decrease in resident macrophages (127). Could PC abnormalities also affect the LC ecological niche? Further studies are awaited.

### 4.3 Fibroblasts

The cellular composition of the dermis includes fibroblasts as well as a host of immune cells such as macrophages, immature dendritic cells, mast cells, and resident memory CD4^+^ T cells (2). Fibroblasts amplify the inflammatory response of the skin (128, 129), promoting type 2 immunity and pruritus in atopic dermatitis (129, 130). The absence of PC on fibroblasts increases the secretion of the proinflammatory cytokine IL-6, which in turn inhibits PC formation on fibroblasts. This feedback loop of PC loss and IL-6 upregulation amplifies the inflammatory response (18).

Inflammatory cytokines and chemokines produced by dermal fibroblasts have a key inflammatory role in the skin (131, 132). Many chemokines originating from fibroblasts bind to receptors expressed by myeloid and T cells, promoting tissue inflammation via immune cell recruitment (128). In addition, fibroblast growth factor (FGF) expressed by fibroblasts can induce KC hyperproliferation (133). Psoriasis is an inflammatory skin disease characterized by epidermal KC overproliferation and abnormal differentiation (134). In psoriatic skin, mRNA levels of FGF and its receptor (FGFR) are elevated (135). High FGF expression is localized in the dermis, and that of FGFR is localized in the basal and suprabasal layers of the epidermis, coinciding with the localization of fibroblasts and KCs (135). The increased FGFR-mediated signaling contributes largely to the epidermal hyperplasia of psoriasis (128). FGF/FGFR signaling has been found to act through PC and can control ciliogenesis while crosstalking with the PC-associated Hh pathway (136). Sustained activation of pathological FGF/FGFR signaling results in primary cilium shortening and inhibition of the Hh pathway; and, the shortening of cilia is associated with a reduction in the speed of the IFT affected by FGF signaling (137). Thus, PC are likely to be the sites of important mediators and effectors of fibroblast inflammation (**Figure 2**).

### 4.4 Skin barrier

PC in KCs are important for the adequate formation of the skin barrier. The cilium structural gene kinase family member 3A (KIF3A) is required for skin barrier homeostasis, and KIF3A deficiency leads to atopic dermatitis susceptibility (138).

Filaggrin (FLG) and loricrin (LOR), two major proteins expressed by KCs during terminal differentiation (139), are major components of the epidermal barrier (140). FLG and LOR deficiencies drive the deterioration of the skin barrier and skin immune dysfunction, important pathogenic factors mediating susceptibility to inflammatory skin diseases (141–143). Skin LOR and FLG expressions correlate with that of PC. Epidermal hyperproliferation due to centrosome amplification results in a significant loss of PC accompanied by a decrease in FLG and LOR expression (14). By contrast, LOR expression downregulation in atopic dermatitis and psoriasis is accompanied by an increase in PC (9, 16). In addition to the reduced FLG expression due to FLG gene mutations, the Th2 cytokine milieu in patients with atopic dermatitis can itself contribute to acquired FLG deficiency (144, 145). Whether this is related to increased PC in response to Th2 stimulation remains to be explored (16). However, FLG expression levels in atopic dermatitis epidermis do not seem to correlate with the percentage of ciliated cells (9), and psoriatic skin samples failed to demonstrate colocalization of PC and LOR (16). Thus, further research is needed to obtain more conclusive evidence.

Airborne particulate matter induces oxidative skin stress, leading to skin barrier dysfunction or immune dysregulation, important contributors to the progression of skin inflammation (146). PM2.5 stimulation inhibits KC differentiation as well as ciliogenesis by activating the c-Jun pathway, which leads also to ciliary length shortening (147). Moreover, high levels of environmental PM2.5 can downregulate the levels of FLG degradation products in the skin and inhibit the expression of skin barrier protein genes such as FLG and LOR in human epidermal primary keratinocytes *in vitro* (145). In dermal fibroblasts, PM2.5 similarly decreases both ciliated cells and cilium length, and induces the generation of excessive reactive oxygen species and the activation of the JNK pathway, promoting localized inflammatory responses (22). The above suggests that PC are also involved in the deterioration of skin inflammation by external environmental stimuli (**Figure 2**).

## 5 Regulation of differentiation and expression of skin immune cells by primary cilia

Lymphocyte subpopulations drive distinct response patterns in the skin. In response to specific skin microenvironmental stimuli, different lymphocyte subpopulations differentiate from common naïve precursor cells and secrete specific cytokines to perform their functions (3). Immune imbalances in T-lymphocyte subsets are important contributors to the disparate phenotypes of inflammatory skin diseases that can be classified according to the specific T-lymphocyte subsets that dominate the response. Examples include the T1 cell-dominated allergic contact dermatitis (148) and vitiligo (2), T2 cell-dominated atopic dermatitis, and T17/T22 cell-dominated psoriasis (2). The effector mechanisms of each T cell subset determine molecular changes in local tissue cells, leading to specific microscopic and macroscopic skin alterations (2).

Hematopoietic cells lack a primary cilium (149), but the PC on other skin cells can still influence the local immune response via several direct or indirect channels:

First, cilia-associated proteins have been shown to be expressed in T lymphocytes, and there is a high degree of morphological and functional similarities (IFT components, vesicular trafficking, and signaling such as Hh) between the immunological synapse (IS) of T lymphocytes and PC, suggesting that the IS may serve as a functional primary cilium homologue (150, 151). Ciliary protein deletions severely affect T cell differentiation, maturation, and immune function. IFT20 knockdown leads to IS assembly defects and polarized TCR recycling disruption in T cells, resulting in impaired TCR/CD3 clustering and signaling on IS (152). During early T cell developmental stages, IFT20 deletion can attenuate inflammatory manifestations by inhibiting T cell development and expression of key inflammatory factors (153). Deletion of the thymocyte-intrinsic ciliary kinesin Kif7, a negative regulator of Hh pathway activation, increases the activation of the pathway, which regulates T cell activation and TCR signaling intensity. However, Kif7 deletion may also decrease the sensitivity of thymocytes to Shh (115). Kif7-deficient thymi reduce the differentiation of CD4^-^CD8^-^ double negative cells to CD4^+^CD8^+^double positive (DP) cells and selectively delay the maturation of DP cells to the CD8 single positive lineage during T cell development (115). Thymocyte-intrinsic Kif7-deficiency results in reduced expression of the surface antigen CD5, which correlates with the intensity of the TCR signal, on DP and mature CD4 and CD8 cells (115).

**Figure 3:**
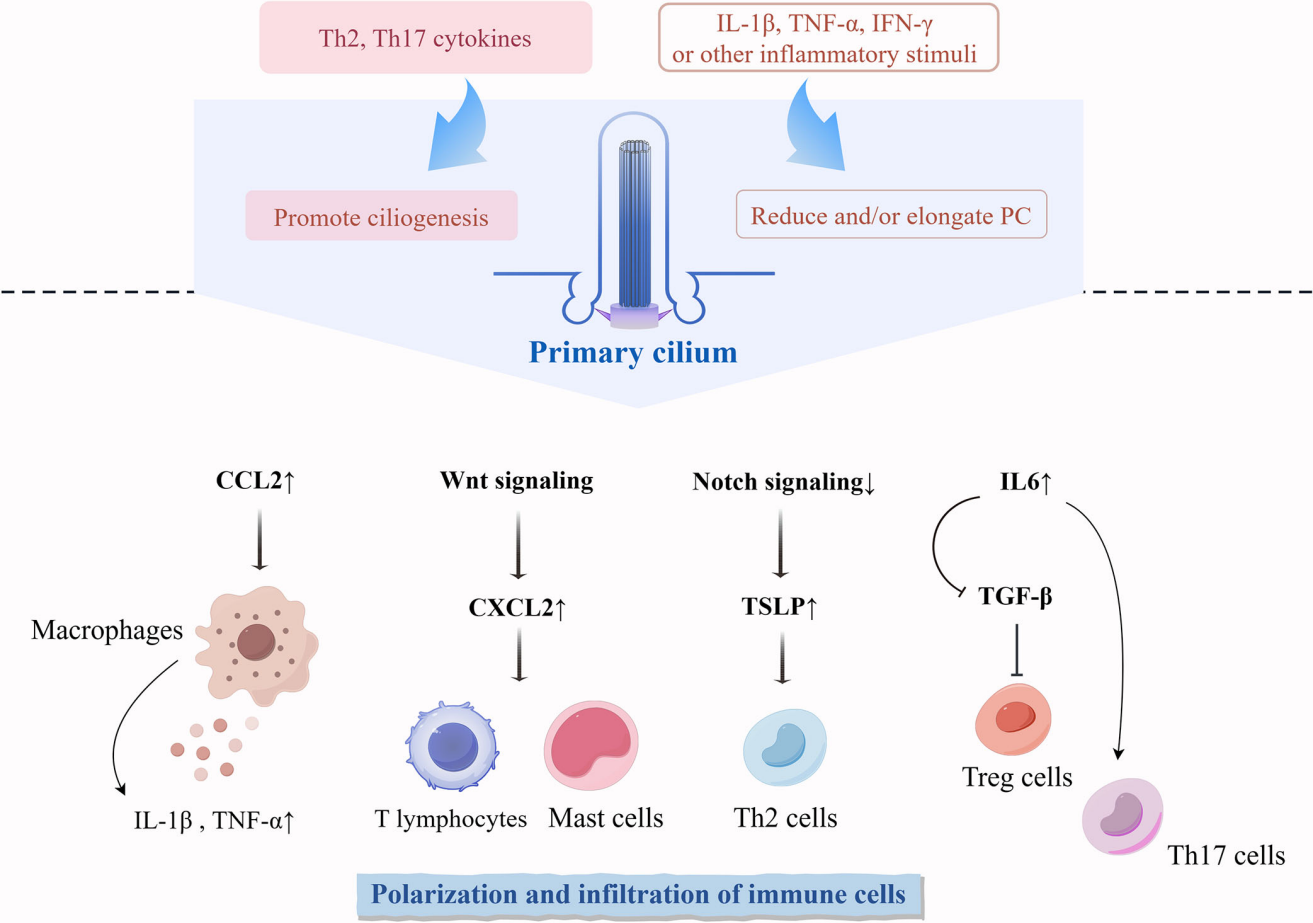
Differentiation and functional regulation of immune cells influenced by PC in the skin. Cytokines during skin immune processes regulate the phenotype of PC on cells. Abnormal expression of PC regulates the polarization and infiltration of non-ciliated immune cells. TSLP, thymic stromal lymphopoietin.

Second, ciliated cells can influence tissue homeostasis by modulating immune cells (**Figure 3**). PC are shown to tightly regulate the niche of macrophages and trigger localized inflammatory responses. Dysfunctional PC can elevate the expression of the macrophage-recruitment chemokine CCL2, leading to the recruitment of mononuclear phagocytes and activation of macrophages, which in turn increases the expression of the pro-inflammatory cytokines TNF-α and IL-1β (127, 154). High expression of CXCL12 in the epidermis and dermis has been shown to induce an increased inflammatory infiltration of immune cells, such as T lymphocytes and mast cells (155). It has been suggested that PC may be able to regulate the expression of CXCL12 through WNT signaling to maintain the homeostasis of the immune and hematopoietic systems (156). The absence of skin-specific Notch signaling causes epithelial cells to secrete large amounts of thymic stromal lymphopoietin, a molecule triggering type 2 immunity and leading to massive inflammation and atopic dermatitis-like disorders (105, 157). The absence of PC on fibroblasts increases the secretion of the pro-inflammatory cytokine IL-6 (18). IL-6-activated inflammation inhibits TGF-β-induced differentiation of naïve T cells to Treg cells and instead activates a pro-inflammatory T cell response dominated by TH17 cells (158, 159).

Finally, various inflammatory microenvironmental stimuli induce alterations in the number and length of PC (**Figure 3**). The stimulation of KCs using Th2 and Th17 cytokines significantly increased the number of ciliated cells (16). Ciliogenesis in primary KCs does not increase in response to stimulation by proinflammatory factors (IL-1β, TNF-α, and IFN-γ) until a certain concentration of proinflammatory factors is reached, increasing levels beyond that threshold gradually decrease the percentage of ciliated cells (16). In inflammatory diseases of other tissues and organs, proinflammatory signals usually reduce and elongate PC. IL-6 stimulation reduces the number of PC on colonic fibroblasts (18). Ciliogenesis is inhibited in acute and chronic pancreatitis tissues (160, 161). After stimulation with proinflammatory cytokines (IFN-γ and TNF-α), thyrocytes significantly reduce ciliogenesis but increase ciliary length (162). IL-1β significantly stimulates ciliary elongation in chondrocytes and fibroblasts, and this elongation drives downstream inflammatory responses in the form of chemokine release (17).

## 6 Conclusions and perspectives

The role of PC in regulating immune system homeostasis has not been widely appreciated, partly due to their absence in differentiated myeloid or lymphoid cells (149). Recent perspectives suggest that PC signaling facilitates communication between ciliated cells and non-ciliated immune cells (23, 154, 156). Additionally, PC and their mediated signals have been implicated in immune processes associated with various skin lesions (9, 13, 16, 30, 33). However, the mechanisms by which PC regulate cellular functions and tissue immune homeostasis via signal transduction are far more complex than previously understood.

The signal transduction function of PC can be achieved in different ways. For example, extracellular vesicles budding from the ciliary membrane transport signaling proteins (25, 163), and their role should not be overlooked. Epigenetically, micro-RNAs (miRNAs) regulate the assembly and genesis of PC and influence the level of cilia-associated signaling (164–168). Whether the aberrant expression of miRNAs in inflammatory skin diseases (83, 169–171) interferes with primary cilium expression to promote inflammation also deserves further investigation.

Simultaneous complex stimuli have different driving effects on primary cilium expression and relevant downstream signaling pathways, but they collectively affect the roles of PC in disease. For example, the expression of ciliary genes (ENO4, INTU, KIF27, PACRG, and STK36), stimulated by the inflammatory factors IFN-γ and TNF-α, is increased but ciliogenesis is inhibited, whereas the high expression of specific miRNAs (miR-146b-3p, miR-21-5p, and miR-6503-3p) decreases both the ciliary gene expression and primary cilium numbers (162). Both inflammatory factors and specific miRNAs synergistically result in shorter and fewer PC in disease (162). The skin is a large and complex organ, and primary cilium-expressing cells in the dermis and epidermis differ somewhat in their sensitivity to specific inflammatory environments to the extent that both the trend of primary cilium expression as well as its immunity drivers may be cell-specific. The characteristic expression of PC in different skin environments cannot be analyzed from a single perspective.

Among the PC-mediated signaling pathways, the transduction of Hh signaling must occur via PC, whereas other signals are only partially dependent on PC (35). In addition, the primary cilium-dependent crosstalk among various signaling pathways is important. Co-localization within the PC lays the foundation for synergistic and crosstalk patterns among some intracellular signaling pathways (29). For instance, PC offer a unique platform for G-protein-coupled receptor signaling to regulate Hh signaling (172, 173). Hh signaling depends on PC; at the same time, PC allow crosstalk between Hh and other signaling pathways such as mTOR (174, 175), RTK (176), and prostaglandin (177) pathways. PC are also able to antagonize the conversion of Hh to the Ras/MAPK pathway (32). The simultaneous inter-pathway coordination via PC is also an important process maintaining the normal functioning and length of the cilia to stably direct downstream signals (177). Multiple signal pathways may co-coordinate immune responses via PC. The characteristic expression of various types of immune signals in skin diseases is the joint result of regulation by the PC and other parties, and the extent to which PC have specific regulatory roles in the pathogenesis of these diseases deserves to be further investigated.

Researchers studying PC usually generate aberrant primary cilium expression models by mutating or deleting the genes of various ciliary proteins. The models help validate the functional significance of the PC and display any independent roles of the ciliary proteome (12, 67). Some scholars have suggested that the function of ciliary proteins during inflammation goes beyond their canonical ciliary function. These researchers propose that PC themselves may not be involved in the transmission of early inflammatory signals, and that the regulatory function of the ciliary proteins in cellular inflammation may be independent of the ciliary axoneme (178). Therefore, whether the regulatory role of some ciliary proteins in downstream cellular responses to inflammation is independent of fundamental primary cilium structures, such as the ciliary axoneme, needs to be further clarified.

We reviewed the functions of major PC signaling pathways and ciliated cells in the skin, examining the potential immunomodulatory effects of PC in the skin and their molecular mechanisms. This paper compiles evidence from various cells, tissues and disease models, providing valuable references for investigating the potential mechanisms by which PC communicates with the immune system in the skin. However, this evidence does not fully represent the specific mechanisms of PC within the skin’s immune environment, highlighting a limitation of this review and uncerscoring the need for further molecular studies. In addition, not all PC in the body are created equal (29). The specificity of PC expressed by diverse skin cells under the different immune environments of skin diseases also needs to be clarified in future studies. What immune signals stimulate PC in skin cells, and what immune pathways and cellular responses do PC drive in response to these stresses? These questions merit deeper exploration. A thorough understaning of the molecular mechanisms underlying PC-mediated immunomodulation in the skin will enrich our understanding of skin physiology and related diseases.
